# Soil compaction reversed the effect of arbuscular mycorrhizal fungi on soil hydraulic properties

**DOI:** 10.1007/s00572-024-01153-9

**Published:** 2024-05-29

**Authors:** Püschel David, Rydlová Jana, Sudová Radka, Jansa Jan, Bitterlich Michael

**Affiliations:** 1 Department of Mycorrhizal Symbioses, Institute of Botany of the Czech Academy of Sciences, Zámek 1, Průhonice, 252 43 Czech Republic; 2 Laboratory of Fungal Biology, Institute of Microbiology of the Czech Academy of Sciences, Prague 4, Vídeňská, 1083, 142 00 Czech Republic; 3 Albrecht Daniel Thaer-Institute for Agricultural and Horticultural Sciences, Division Urban Plant Ecophysiology, Humboldt-Universität zu Berlin, Lentzeallee 55/57, Berlin, 14195 Germany

**Keywords:** Arbuscular mycorrhizal fungi, Irrigation, Pot shape, Sand–zeolite–soil mixture, Tomato, Water holding capacity

## Abstract

Arbuscular mycorrhizal fungi (AMF) typically provide a wide range of nutritional benefits to their host plants, and their role in plant water uptake, although still controversial, is often cited as one of the hallmarks of this symbiosis. Less attention has been dedicated to other effects relating to water dynamics that the presence of AMF in soils may have. Evidence that AMF can affect soil hydraulic properties is only beginning to emerge. In one of our recent experiments with dwarf tomato plants, we serendipitously found that the arbuscular mycorrhizal fungus (*Rhizophagus irregularis* ‘PH5’) can slightly but significantly reduce water holding capacity (WHC) of the substrate (a sand–zeolite–soil mixture). This was further investigated in a subsequent experiment, but there we found exactly the opposite effect as mycorrhizal substrate retained more water than did the non-mycorrhizal substrate. Because the same substrate was used and other conditions were mostly comparable in the two experiments, we explain the contrasting results by different substrate compaction, most likely caused by different pot shapes. It seems that in compacted substrates, AMF may have no effect upon or even decrease the substrates’ WHC. On the other hand, the AMF hyphae interweaving the pores of less compacted substrates may increase the capillary movement of water throughout such substrates and cause slightly more water to remain in the pores after the free water has drained. We believe that this phenomenon is worthy of mycorrhizologists’ attention and merits further investigation as to the role of AMF in soil hydraulic properties.

## Introduction

The role of arbuscular mycorrhizal fungi (AMF) in plant nutrition is well established (Smith and Read 2008), whether in the uptake of phosphorus (Jansa et al. 2011; Smith et al. 2011) or nitrogen (Bücking and Kafle 2015; Hodge and Fitter 2010; Hodge and Storer 2015; Johansen et al. 1992). How AMF affect plant water relations also has been studied extensively, as shown in the review by Augé (2001) and numerous references therein. In our own research, we brought these two topics together and found that mycorrhizal pathways of phosphorus and nitrogen uptake prevail under water deficit (Püschel et al. 2021, 2023). A recent review by Abdalla et al. (2023) summarizes our current understanding as to the role of AMF in improving plant water status under drought, including the biophysical mechanisms involved in water fluxes within soil pores to roots and through plant tissues to stomata. While it remains controversial whether the provided amount of water translocated by AMF (and particularly inside their hyphae) is physiologically meaningful (Faber et al. 1991; George et al. 1992; Kakouridis et al. 2022; Püschel et al. 2020), there is no doubt that some role of AMF in water translocation exists, either direct (within the hyphae) or indirect (wicking along the hyphal surface) (Allen 2007; Kakouridis et al. 2022).

Transpiration-driven water fluxes from soil to plants are affected by soil hydraulic properties, specifically soil water retention and hydraulic conductivity (Abdalla et al. 2023). While these two soil parameters primarily depend on particle size distribution (soil texture) and spatial particle arrangement (soil structure), both water retention and hydraulic conductivity also have been found to be altered by the presence of AMF. The moisture retention properties of a ‘mycorrhizal soil’ were probably first pointed out by Augé et al. (2001) and Neergaard Bearden (2001). Although some further evidence of AMF effects on soil water retention and hydraulic conductivity has recently emerged (Bitterlich et al. 2018a, b; Pauwels et al. 2020, 2023), this is merely due to an effort by one group of researchers rather than to a trend reflecting general awareness of the issue. Whether, in what context, and to what extent AMF affect soil hydraulic properties remain poorly understood and thus largely unresolved.

In one of a series of experiments that we have conducted in recent years, focused on various aspects of the functioning of arbuscular mycorrhizal symbiosis under soil water deficit, we serendipitously noticed that AMF can have a small but significant effect on water holding capacity (WHC) of the substrate. This was taken into account in subsequent experiments wherein we also strove to quantify the WHC of mycorrhizal and non-mycorrhizal pots pursuing a specific irrigation approach that can be termed “saturation cycles.” These cycles were repeated at approximately weekly intervals and with a high number of replicates. The datasets revealed some interesting patterns that we believe are worth sharing with others.

## Materials and methods

### Common aspects of both experiments

WHC was quantified in two greenhouse experiments, hereafter referred to as Exp-A (not published) and Exp-B (Püschel et al. 2023). Both experiments were conducted in the same substrate (see below), with the same model plant species (dwarf tomato – *Solanum lycopersicum*, cv. ‘Micro Tom’), and inoculated with the same AMF isolate (*Rhizophagus irregularis* ‘PH5’). For details on mycorrhizal inoculation and soil microbial filtrate preparation, we refer the reader to Püschel et al. (2023) and the corresponding Supplementary Information. The protocol was very similar for both experiments, unless explicitly stated otherwise. Both experiments also shared the same growing facility (greenhouse with supplemental lighting from high-power LED panels) as described in the above referenced article. Exp-A was conducted in the spring of 2021 and Exp-B followed in summer of the same year.

In the early stage relevant to this report on WHC, both experiments had only two main treatments: Half of the pots were mycorrhizal (hereafter referred to as ‘M’), while the other half were non-mycorrhizal (‘NM’) controls. There were 50 replicates per treatment in each experiment.

### Preparation of substrate and filling of pots

**Fig. 1:**
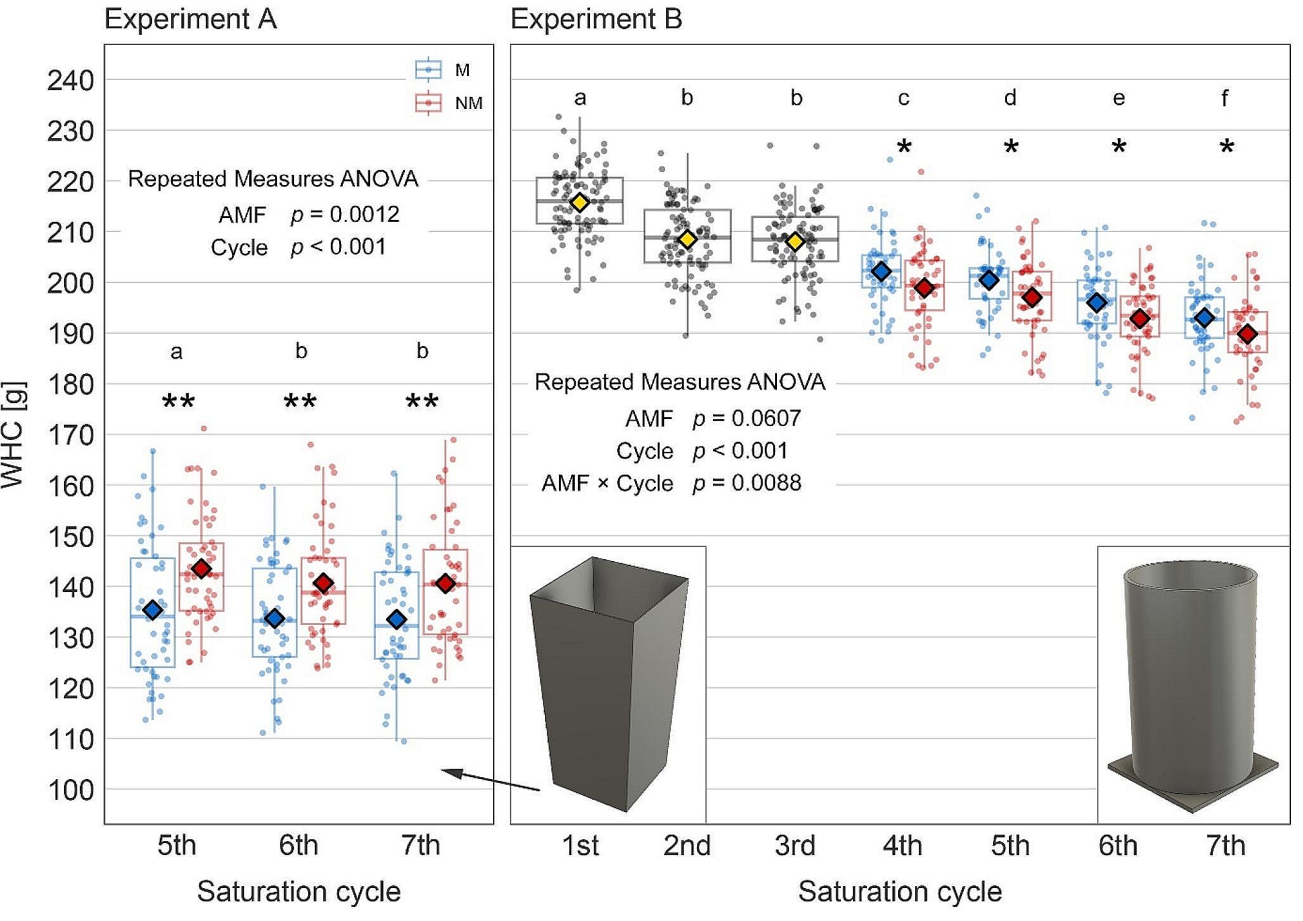
Water holding capacity (WHC) of 1 kg of dry substrate – sand-zeolite-soil mixture, 9:9:2 (v: v:v) – observed in Experiment **A**, conducted in pots with tapered walls, and Experiment **B**, conducted in pots of cylindrical shape. The effects of the experimental factors – presence of arbuscular mycorrhizal fungi (AMF) and saturation cycle (Cycle) – are shown according to repeated measures ANOVA. Different letters indicate significant differences in WHC between the saturation cycles according to Tukey’s HSD test. For the individual saturation cycles for which a *t*-test identified a significant effect of *Rhizophagus irregularis*, the boxplots and superimposed points are separated by color, with blue representing mycorrhizal (M) and red control (NM) treatments; asterisks indicate a level of significance according to the *t*-test (* 0.01 ≤ *p* < 0.05, ** 0.001 ≤ *p* < 0.01). Center lines of the boxplots indicate the medians, box limits represent the 25th and 75th percentiles and whiskers extend to 1.5 times the interquartile range. Diamond shape points indicate treatment means. In cases of non-significant difference, M and NM treatments are plotted together in grey and the mean in yellow. Horizontal variation within each category (*n* = 50 per treatment) is determined randomly to separate the points

Exp-A was conducted in standard plastic pots with a square footprint and tapered walls (bottom 8.5 × 8.5 cm, top 10.2 × 10.2 cm, height 21 cm; sketch included in Fig. 1A) with several factory-made drainage holes at the bottom. Its 2 L volume was partially reduced by a layer of surface-sterilized marble stones (approximately 1.2–2.0 cm in diameter) placed at the bottom, so that only 1.6 L of substrate was available for plant growth. A plastic mesh on top of the stones prevented substrate from leaking into the stone layer. The stone layer provided drainage and also reduced the substrate depth to that of a moisture meter probe so there was no zone of substrate outside its measurement range.

Exp-B was conducted in pots of our own design, made of thick-walled PVC-U tubes cut to length and glued to square plates with four drainage holes. The resulting cylindrical pot with a volume of slightly more than 2 L (inner diameter 11.7 cm, height 20 cm; sketch included in Fig. 1B) had rigid, straight walls. The stones were used for the same reason as in Exp-A, such that 1.7 L of substrate was placed in the pots of Exp-B.

Both experiments were performed using the same substrate consisting of autoclaved sand, autoclaved granular zeolite, and γ-sterilized (> 25 kGy) soil thoroughly mixed in ratio of 9:9:2 (v: v:v). See Püschel et al. (2023) for details on preparation and physicochemical properties of the substrate and Püschel et al. (2021) for its water retention curve. Special care was taken to fill the pots evenly within each experiment (i.e., with the same amount of substrate compressed to the same bulk density). The substrate, slightly moistened after autoclaving the sand and zeolite, was poured in three successive batches into a plastic measuring cylinder cut to the 1.6 L (or 1.7 L) mark and compacted with three free-fall strokes of a 750 g piston. The cylinder was levelled with a knife to remove excess substrate and to achieve exactly 1.6 L (or 1.7 L, for Exp-B) volume. The substrate was then poured into ziplock bags and weighed. To determine the dry weight of the substrate, substrate in several additional bags was air-dried and the relationship between fresh and dry weights was determined. This allowed the dry weight of the substrate in each individual pot to be retrospectively estimated with sufficient accuracy for WHC calculation.

In order to achieve a similar bulk density of the substrate in each pot, the pots were filled by one person using that individual’s best efforts to press the substrate evenly by hand. The initial bulk density achieved in the pots of the two experiments was estimated to be approximately 1.18 and 1.16 kg per liter of dry substrate, respectively, providing similar hydraulic properties in all pots.

### Plant cultivation and method of irrigation

The substrate for both experiments was fertilized with micronutrients as described in Püschel et al. (2023). In addition, the plants were fertilized throughout the course of the experiments to avoid nutrient limitation of the plants. Because the Exp-A concept required healthy and sufficiently nourished plants, a commercial fertilizer, Kristalon Gold (AGRO CS, Říkov, Czech Republic), was used based on previous extensive experience in our system. The 210 mL total amount of application solution per pot provided 317.5 mg N, 105.8 mg P, 335.2 mg K, and 405.7 mg S. In the case of Exp-B, controlled fertilization was required to avoid excess nutrients, especially nitrogen, so it was fertilized using Long Ashton nutrient solution providing 112.51 mg N, 27.45 mg P, 139.32 mg K, and 32.34 mg S per pot. It is likely that some of the nutrients were lost from the pots in both experiments as a result of draining of excess water (see below).

Initially, young plants were watered as needed to promote successful establishment and growth. Later, we used what we call “saturation cycles” as the primary method of irrigation. We wanted to supply the entire volume of the substrate with an abundance of water for a sufficient period of time so that it could penetrate into most pores, similar to the situation in nature after a long or intense rainfall. In Exp-A, this was achieved by placing the pots into large plastic boxes, which were then slowly filled with water (the design of drainage holes in these pots precluded any kind of bottom sealing) until the water in the boxes was level with the substrate surface. Separate boxes were used for M and NM pots to avoid cross-contamination. In Exp-B, individual pots were placed on a microporous rubber pad that sealed the four drainage holes in each pot, then the pots were watered from above (a repeated gentle shower) until water appeared on the surface. In both experiments, the pots were allowed to saturate for 30 min. The pots were then removed from the bath or rubber pads, respectively, and the excess water was allowed to drain freely from the pots. One hour later, when no more water was dripping from them, the pots were weighed. The weight of the empty pots (with all accessories), the dry weight of the substrate, and the fresh biomass of the plants (estimated from photos as a percentage of the final fresh plant biomass determined at harvest) were subtracted. To this end, plant fresh weight measured at final harvest was multiplied by 0.5, 0.65 and 1 for cycles 5, 6 and 7, respectively, in Exp-A. In Exp-B, the coefficients to multiply final fresh weight (excluding fruits) harvested from each pot to estimate plant weight at different time points were 0, 0.025, 0.15, 0.3, 0.6, 0.7, and 0.8 for the 7 sequential saturation cycles. The resulting water contained in the pots was recalculated relative to 1 kg of dry substrate to allow direct comparison of the two experiments.

### Harvest and data analyses

Plant biomass was harvested and mycorrhizal root colonization quantified as presented in Püschel et al. (2023). In addition, to describe the morphological characteristics of the roots, about half of the roots were spread in water within a transparent plastic tray and scanned at 600 dpi on a flatbed scanner with two-sided illumination (Epson Perfection V800 Photo). The images were analyzed for root morphology using WinRhizo software (Regent Instruments, Quebec City, Canada), and the data were recalculated for the entire root system. Importantly, because Exp-A was harvested 2–7 days after the last saturation cycle, the biomass data obtained are well relevant to the WHC records, especially from the final, 7th, cycle. In the case of Exp-B, the main phase of the experiment started only after the last saturation. Plants were harvested 29–31 days later, so biomass and root morphology are not in this case so relevant for the period of WHC quantification as in Exp-A. The length of extraradical mycelium (ERM) also was quantified in both experiments using a membrane filtration technique (Jakobsen et al. 1992), modified as described previously (Püschel et al. 2007).

The data were analyzed statistically in RStudio Team, version 2023.03.0. (RStudio: Integrated Development Environment for R. RStudio, PBC, Boston, MA URL. http://www.rstudio.com/), and graphs were generated using the ggplot2 package. Prior to analysis, data were tested for normality (Shapiro–Wilk test) and homogeneity of variances (Levene’s test). Differences in plant biomass or root morphology between the M and NM treatments were analyzed by *t*-test. WHC data were analyzed using repeated measures ANOVA with inoculation as a categorical factor and saturation cycles as time points. Tukey’s HSD test was used to separate means when comparing WHC between cycles. A *t*-test was then used to determine at which cycles WHC differed significantly between the M and NM pots.

## Results and discussion

It is important to mention at the outset that the mycorrhizal symbiosis was sufficiently developed in the M treatments of both experiments, while AMF were absent in the roots and substrates of the NM treatments. In Exp-A, mycorrhizal structures (hyphae, arbuscules, or vesicles) were present in 26% of the root length (on average) and there was a mean of 321 mm of ERM per gram of dry substrate. In Exp-B, 65% of root length was colonized (see Püschel et al. (2023) for more details on colonization) and there was a mean of 582 mm of ERM hyphae per gram of dry substrate. This difference in mycorrhiza development, particularly in root colonization, can be explained by the amount/composition of fertilizers applied. Because the Kristalon Gold fertilizer provided a surplus of nutrients compared to the Long Ashton solution, the mycorrhizal pathway of nutrient uptake was probably less important and, consequently, mycorrhizal colonization of roots or substrate was less intense in Exp-A as compared to Exp-B. In both experiments, colonization was quite variable among plants, which is our consistent finding with this dwarf tomato cultivar. Relatively less intensively developed ERM networks also might be attributed in part to the generally low response of this plant species to AMF (our own long-term observation) and partly to less ERM produced by the *R. irregularis* isolate compared to other isolates (Sudová et al. 2010). Total dry weight of plants was 5.32 g (M) and 5.68 g (NM) in Exp-A and 2.0 g (M) and 2.2 g (NM) excluding fruits or 4.0 g (M) and 4.6 g (NM) including fruits in Exp-B.

In Exp-A, AMF were found to *decrease* WHC of the substrate **(**Fig. 1A**)**. As mentioned above, this was a serendipitous discovery made after the pots of well-established M and NM plants were first weighed out of curiosity after the 5th saturation cycle (quantification of WHC was originally beyond the scope of the experiment). This was then repeated for the last two saturation cycles. When plant biomass was quantified after the harvest, the calculations were then updated with a better representation of the plant weight relevant to the time of a given WHC quantification. Although the data had a rather high variability and the difference between means of the M and NM treatments was on the order of units of grams of water per 1 kg of dry substrate, it was statistically significant (*p* < 0.01). Furthermore, it was consistent in all three saturation cycles 5th through 7th **(**Fig. 1A**)**.

Although it could be admitted that any recorded differences in WHC between M and NM substrates might stem from possible effects of the mycorrhizal fungus on morphology or even just quantity of plant roots (Berta et al. 1995; deVries et al. 2021; Hetrick 1991), our results from Exp-A, for which we have root data relevant to the time of WHC quantification, do not support such a notion. None of the root parameters measured differed significantly between the M and NM plants: fresh root weight was 12.14 g (M) vs. 12.7 g (NM), *p* = 0.1106; dry root weight was 1.37 g vs. 1.38 g, *p* = 0.7822; root length was 203.0 m vs. 196.3 m, *p* = 0.6097; root surface area was 3375 cm^2^ vs. 3380 cm^2^, *p* = 0.9823; and root volume was 44.8 cm^3^ vs. 46.6 cm^3^, *p* = 0.5424.

Based on the above finding, Exp-B was set up with quantification of WHC as a secondary objective that could be achieved during the initial phase of the experiment. WHC was quantified from the beginning, so records were obtained for all seven saturation cycles applied. For the first three cycles (days 9, 17, and 23), no effect of AMF on WHC was found (Fig. 1B). This is not surprising, as the plants were still very small and the mycorrhizal symbiosis was still in its early stage of development (with the external mycelium only starting to develop in the substrate). However, a significant *positive* effect of AMF on WHC (i.e., directly the opposite of that in Exp-A) was observed from the 4th cycle onwards (days 29, 36, 43, and 49; Fig. 1B), when the mycorrhizal fungus networks arguably were present in the substrate. We were curious to see if WHC would correlate with length of the ERM. Such correlation was far from significant, however, in either experiment (*R*^*2*^ = 0.01, *p* = 0.5 and *R*^*2*^ = 0, *p* = 0.98, respectively). It is possible that because of the combination of a mycorrhiza-unresponsive host plant, mycelium traits of the specific *R. irregularis* isolate used, and mineral fertilization, there were relatively few external hyphae in our experimental system. The variability in ERM length therefore was not a meaningful continuous explanatory variable for the WHC. Thus inoculation simply was a 2-level categorical variable comparing the effect of the presence of mycorrhizal fungus hyphae with their absence.

The comparison of WHC data from the two experiments reveals two interesting findings that pose two important questions. First, why was the observed effect of the mycorrhizal fungus on substrate WHC directly opposite in the two experiments (negative in Exp-A, positive in Exp-B)? Second, why was the substrate in Exp-A able to hold significantly less water (by about 60 g per 1 kg dry substrate) than that in Exp-B after the same number of saturation cycles (Fig. 1)? It is obvious that there had to be another factor involved, because the mere presence or absence of AMF cannot explain these contrasting results of the two experiments. What might this other factor be?

An indication for the answer may be found in the results of Exp-B, from which we have data on WHC over a prolonged period, including seven repeated measurements. It is noteworthy that WHC decreased significantly over time by more than 20 g·kg^− 1^ (Fig. 1B). We are quite confident that this was a consequence of saturation cycles during which the substrate particles were first lifted by the upward pressure of the water (buoyancy) and then subsided as the free water drained from the pots and the substrate profile dried over the following days. We observed, but unfortunately did not quantify, a slight subsidence of the substrate during the course of both experiments. This movement probably caused added compaction of the substrate because of rearrangement of substrate particles and changes in pore size and distribution. The result was that, with each saturation cycle, the same amount of substrate was holding slightly, but measurably, less water than before.

**Fig. 2:**
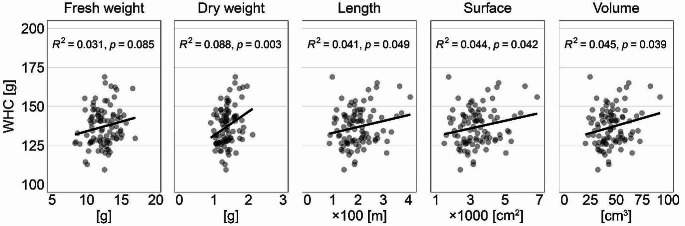
Correlation between the water holding capacity (WHC) of 1 kg of dry substrate in the 7th saturation cycle and the morphological characteristics of the roots in Exp-A. Because the *t*-test did not reveal significant differences between the root characteristics of mycorrhizal and control plants, both treatments are plotted together, not distinguished by color. Linear regression lines are shown together with *R*^*2*^ and *p* values

Was the lower WHC in Exp-A compared to Exp-B caused by bigger plants with more roots (1.38 g vs. 0.84 g of dry roots, respectively), possibly increasing substrate drainage? Our data does not support this notion. We correlated WHC in the last saturation cycle of Exp-A with all measured root parameters to test whether the ability of the substrate to hold water was inversely proportional to any of them. While the regressions generally explained little variability, the correlations were either not significant (fresh weight) or significant in the opposite direction: WHC was positively correlated with dry weight, length, area, or volume of the roots (Fig. 2). Thus, the much lower WHC in Exp-A cannot be explained by there being more roots in these pots compared to the pots in Exp-B with generally smaller plants. Further, we consider it highly unlikely that the lower WHC (by ca. 60 g·kg^− 1^) was caused by higher transpiration of bigger plants and faster loss of water from the pots. To put this into context, our records indicate that Exp-B plants required ca. 77 g water *daily* at the end of the experiment. Even if the daily water demand of the larger Exp-A plants was likely higher, the transpiration loss within the narrow window between saturation and WHC quantification was only a fraction of this, especially considering that plants’ need for water was likely more than satisfied during the period of saturation. We thus cannot explain the striking difference in WHC of the substrate found in the two experiments because of differently sized plants.

We propose an explanation that answers both of the aforementioned questions. The difference in WHC between the experiments as well as the opposite effect of AMF were related to the different shape of the pots. Although the up and down movement of the substrate was observed in both experiments, we think that its impact was stronger in pots with tapered walls (Exp-A), where the substrate was gradually squeezed into a narrower space and thus became compacted even more than in the cylindrical pots (Exp-B). This could have several important consequences. First, repeated mechanical disturbance through substrate particle movement and subsidence upon saturation cycles could slow down intensive substrate colonization by the ERM. For instance, such disturbance-related effects have been reported previously for tillage (Brito et al. 2011). Second, the compaction could have resulted in slightly different pore size distribution and had negative consequences for diffusion of gaseous oxygen which would be limited through fine and disconnected pores. Both scenarios could have reduced the development of ERM and thus also have affected how the mycorrhizal fungus contributed to forming soil structure by enmeshment of soil particles or production of compounds changing soil water repellency that are referred to as ‘glomalin’ in the literature (Rillig et al. 2010; Rillig and Steinberg 2002). It is possible that more developed hyphae in Exp-B than in Exp-A exerted pronounced effects on formation of substrate structure and thereby counteracted compaction (Leifheit et al. 2014). Finally, while in Exp-A the fungal structures were probably just another object occupying the narrow pore space, further limiting gas diffusion and possibly also water holding capacity, in Exp-B the same fungal structures, but present in larger pores, could have altered the capillary forces in the substrate enough to allow it to hold significantly more water after the free water had drained away than if such fungal structures were absent. Similar thoughts have been elaborated in several recent publications, e.g., by Marcacci et al. (2022) and Hammer et al. (2024).

### Limitations of the study

Obviously, consistent/reproducible bulk density of the substrate is extremely important in any study relating to soil water. Although we did our best to minimize this problem during the filling of the pots, some of the observed variability in the WHC data may be because of this issue, which we counteracted by a high number of replications (*n* = 50) and repeated measurements. Second, the fresh biomass of the plants to be included in the WHC calculation was not measured directly but was estimated (from the biomass quantified at harvest and its estimated proportion at the time of the given saturation cycle). There is no ideal solution to this problem not having its own drawbacks. Even if a certain number of plants would be destructively harvested after a given saturation cycle, high biological variability means that these fresh-weight records would be fully accurate only for the harvested plants and could not be used with full confidence for the rest of the plants. At the same time, such an approach would reduce the number of replications for subsequent cycles. Finally, because we did not collect and analyze the water that drained from the pots after each saturation cycle was over, we do not know how much (if any) fine substrate particles may have been washed out of the pots and how this might have changed the substrate texture.

## Conclusion

We demonstrated significant effects of AMF presence on WHC that appeared to depend on the structure of the substrate (i.e., the spatial arrangement of substrate particles and pores). Although the measured differences between the means were quite small in absolute terms, they were observed repeatedly and on a large number of pots. Our results suggest that in compacted substrates, AMF may have no effect or may even decrease the substrates’ WHC. On the other hand, in less compacted substrates, interwoven AMF hyphae (or their products) may increase capillarity and slightly more water may then remain in the pores after free water is drained away. As we believe that such findings are unique in mycorrhizal research, we aim to further address the issues described above. Therefore, in the future we plan to use more natural soil and also to experimentally vary soil/substrate texture instead of using just the artificial substrate which predominantly comprised relatively large sand and zeolite particles. From a practical point of view, our results indicate that, in pot experiments, methodological details such as the need to fill the pots to the same bulk density should not be overlooked, as this can significantly affect how much water the system can retain and thus how much soil solution containing the nutrients is actually available to the plants. It also means that mycorrhizologists conducting drought experiments in pots should be aware that their “equal watering regimes” may not result in equal moisture conditions, because the presence of AMF may have effects other than simply “increasing water uptake,” as is often described. Finally, this also could be important for experiments addressing salinity stress or even nutrient uptake, because mycorrhiza-induced changes in water holding capacity can, in a long experiment, change salt accumulation or nutrient leaching differently in M and NM conditions.

## Data Availability

No datasets were generated or analysed during the current study.
